# The Technique of Thiersch’s Method of Skin-Grafting

**Published:** 1895-01

**Authors:** Theodore Dunham

**Affiliations:** New York, Instructor in Surgery in the New York Post-Graduate Medical School


### Selections and Abstracts.

---

The Technique of Thiersch's Method of Skin Grafting. By Theodore Dunham, M. D., New York, Instructor in Surgery in the New York Post-Graduate Medical School.—The elegance of the result in skin grafting depends on nice attention to certain details. The size of the area that can be covered depends largely on deftness in the operation, and largely on careful preparation so that no time may be wasted. In the following brief sketch I have tried to map out the operation as it may be done for covering considerable areas of skin deficiency.

*Preparation of the Wound Surface.—The surface to be grafted should be one of normal tissues.* A recent wound surface, whether from injury or operation, needs only perfect disinfection and then douching with salt solution. A surface of granulations requires more preparation. Wash it every second day with soap and water, and bichloride 1:1,000, and dress with iodoform gauze saturated with balsam of Peru. Dress thus until the surface is level, rosy, and firm. This may require two weeks. A few hours before operation apply a wet bichloride compress.

*Preparation of the Thigh for Removing Grafts.*—The thigh is the most convenient part of the body from which to take grafts. Which thigh is chosen will depend on which side of the table gives the operator the readiest access to the wound to be covered. The day before operation the thigh should be shaved, scrubbed with soap and water and with bichloride, and wrapped in damp bichloride gauze. Shortly before operation wash with salt solution and apply a salt gauze compress, and cover this by two bichloride towels applied as follows: Let the lower edge of one come below the knee, wrap it around the extremity and secure it by a bandage round the knee; lay the upper edge of the other towel at the groin, wrap it around the extremity, and secure it by a bandage passed round the highest part of the thigh. These towels protect the dressing until operation; at operation they are quickly folded back, one up, the other down, the gauze removed, and the skin is exposed, snugly bordered by bichloride towels.

*Instruments, Solutions and Dressings.*—Use a razor ground flat on one side. For an operation of any magnitude there should be two razors, for the edge gradually loses its keenness. A flat retractor with a rounded edge serves well for scraping off granulations. For removing cicatrized borders and scraping irregular surfaces the Volkman spoon is convenient. A probe is an aid to the fingers in adjusting the grafts. A pair of rough bathing mittens should be boiled an hour and placed in salt solution. A quarter of an hour before operation the instruments should be laid in 1:20 carbolic. Before being used they are dipped in salt solution. Throughout the operation the only solution used is one of sodium chloride in water, 6:1,000. Two large tin pails of this solution should be made a few hours before operation and boiled an hour; one should be allowed to cool, the other kept hot. At operation a bowl of this solution will be wanted for sponges, another for the grafts.. These should be kept at body temperature, which can be maintained by mixing from the two reservoirs. There should be a third bowl of salt solution for the hands. For the dressings sterilize loose gauze by soaking it over-night in bichloride 1:1,000. Sterilize four gauze bandages in the same way. Before operation place a portion of each in salt solution. Cut strips of gutta-percha tissue three-quarters of an inch wide and long enough to reach across the wound and lap over on both sides. Wash them with soap and water, soak over-night in bichloride 1:1,000, and place them in a dish of salt solution.

*Position of Patient on Operating Table.*—Place pillows beneath the patient's hips, back and head, and place a rest beneath the leg so that the thigh may form a bridge half a foot above the table. If the area to be grafted is upon an extremity, suspend the extremity vertically.

*Operation.*—Give ether. Surround the field of operation with bichloride towels. Disinfect the hands with especial thoroughness. Scrape off all granulations and cicatrized borders with the edge of the retractor and the Volkman spoon. Wash clean with salt solution, and at once apply a compress of salt gauze and bind it on tightly with a gauze bandage to check oozing. Throw back the towels from the thigh. Let an assistant, with the rough mittens on his hands, grasp the thigh on either side and draw the skin tense. The operator places his left thumb on the skin at one end of the thigh to steady it, lays the razor upon it at a slight angle, makes a sawing motion, and at the same time increases the angle until the razor bites into the skin. This part of the operation is illustrated in the cut. As the sawing motion is continued, a graft is shaved off comprising a third or half the thickness of the skin. It may be made nearly the length of the thigh. The widest grafts are got from the comparatively flat surface over Scarpa's triangle. Place the graft in a dish of lukewarm salt solution. Continue for ten minutes cutting grafts and putting them into the salt solution. Then remove the compress from the surface to be grafted. Oozing will have ceased. Apply the grafts to the surface. They will have curled, the raw side turned in. To uncurl them place one end on the surface to be grafted and uncurl it. Now, by laying a finger on the graft and making a rapid to-and-fro motion, the graft can be made to uncurl continuously along its entire length. Once flat, the graft may be moved to the position desired by the fingers, and more delicate adjustments made by the probe. If the borders of the surface are abrupt, let the grafts run up the borders and lap over the surrounding skin. Let the edges of the grafts lie in apposition or overlap. Thus cover in the whole area. Wash off any blood by letting salt solution trickle gently from a sponge over the grafted area. Now take a strip of the gutta-percha tissue. Lay one end on the sound skin at one side of the wound and place a thumb upon it; then, with a winding motion, lay it across the wound. Let the next strip slightly overlap the first. Apply strips thus slightly imbricated until the whole grafted area is covered. If the area is upon an extremity, take a bandage from the salt solution and make a snug spiral over the gutta-percha tissue. The grafts will now be all in perfect position and secure from disturbance by the further dressings. Wring gauze lightly from the salt solution and apply it. Cover it by a sheet of gutta-percha tissue to maintain moisture. Apply a layer of bichloride gauze and of absorbent cotton and a bandage. Immobilize by splints suitable to the region. On an extremity coaptation splints are the best.

The pearly-white oozing surface from which the grafts have been taken is best dressed, according to Dr. Abbe's recommendation, by covering it with a sheet of gutta-percha tissue.

Beneath this a new epidermis forms in the course of a week, and the dressing need not be disturbed during that time.

*Subsequent Dressings.*—The grafted area should be dressed every second day. The same strict aseptic precautions are observed as at the operation. The gutta-percha strips are removed as follows: Pick up one end of a strip and curl it back sharply upon itself; strip it from the grafts beneath, keeping it all the while sharply curled. By observing this manœuvre all danger of lifting or of shifting the grafts is avoided. Douche the surface with sterilized salt solution. Sponge off the strips of gutta-percha tissue in bichloride solution and rinse them in salt solution. Dress exactly as at the operation. On the second day the grafts will be rosy. Any abrupt margins will have become reduced and the surface be nearly flat. After the tenth day no dressing is required; but it is well to wear a cloth spread with vaseline for a few days longer.

Using the foregoing technique, I have covered an area of seventy-two square inches in two hours, including the dressing. This is at a rate of one minute and three-quarters to a square inch. Smaller areas would require proportionately more time.

